# Investigating the determinants of fear of cancer recurrence in patients with early‐stage non‐small cell lung cancer: Insights from a restricted cubic spline model

**DOI:** 10.1002/cam4.7406

**Published:** 2024-06-22

**Authors:** Man Liu, Lu Liu, Zhuoheng Lv, Yousheng Mao, Yan Liu

**Affiliations:** ^1^ Department of Thoracic Surgery, National Cancer Center, National Clinical Research Center for Cancer, Cancer Hospital Chinese Academy of Medical Sciences and Peking Union Medical College Beijing China

**Keywords:** early‐stage non‐small cell lung cancer, fear of recurrence, influencing factors, restricted cubic splines

## Abstract

**Objective:**

This study aims to investigate the determinants and the dose–response relationship of fear of cancer recurrence among patients with early‐stage non‐small cell lung cancer (NSCLC), aiming to inform prevention and intervention strategies.

**Methods:**

Employing a cross‐sectional design, we analyzed data from 677 postoperative NSCLC patients who received treatment at National Cancer Center between January 2022 and August 2023. Data collection involved a general demographic survey, the Fear of Progression Questionnaire‐Short Form, Hope Herth Index, and Social Support Rating Scale. We employed logistic regression and restricted cubic spline models to identify factors influencing fear of recurrence (FCR).

**Results:**

Univariate regression analysis identified female gender and having minor children as significant risk factors, while being older than 60, a higher income, and elevated levels of hope and social support emerged as protective factors. Multivariate logistic regression revealed age (OR = 0.392, 95% CI 0.205–0.750), monthly income (OR = 0.016, 95% CI 0.315–0.886), hope level (OR = 0.305, 95% CI 0.187–0.496), and social support (OR = 0.584, 95% CI 0.375–0.908) as independent influencers of recurrence fear. The restricted cubic spline model indicated a nonlinear impact of hope and social support on this fear.

**Conclusion:**

Analysis using the restricted cubic spline model underscores the influence of age, income, hope, and social support on FCR, with a nonlinear dose–response relationship evident between hope, social support, and fear. Prioritizing the enhancement of social support before increasing hope levels can rapidly and effectively alleviate FCR.

## 1 INTRODUCTION

The Global Cancer 2020 report by the World Health Organization's International Agency for Research on Cancer revealed that there were approximately 19.3 million new cancer cases worldwide in 2020, with projections indicating an increase to 28.4 million cases by 2040.^1^ Specifically, lung cancer accounted for 2.2 million new cases and 1.8 million deaths in 2020, marking it as the primary cause of cancer‐related mortality globally. Its incidence has been on the rise in recent years.^2^ Notably, non‐small cell lung cancer (NSCLC) represents about 85% of all lung cancer cases.^3^ Advances in molecular targeted therapy and immunotherapy have significantly improved survival rates for NSCLC patients, particularly those with sensitive gene mutations or in advanced stages, effectively reducing metastasis and recurrence rates. Despite these advancements,^4^, ^5^ approximately half of the patients still face a substantial risk of recurrence and metastasis post‐surgery.^6^

The increasing prevalence of health screenings and the routine use of low‐dose spiral CT scans have led to more early‐stage lung cancer detections. According to the 8th edition of the TNM staging system, the 5‐year survival rates for stage 1A and 1B lung cancer patients are 77%–92% and 68%, respectively.^7^ Despite these encouraging survival statistics for early‐stage NSCLC, patients continue to experience significant fear of cancer recurrence (FCR), impacting their quality of life. FCR, defined as the fear or concern about the possibility of cancer recurrence, progression, or metastasis,^8^ is a prevalent and persistent issue among NSCLC survivors.^9^ This psychological challenge, often considered the most common and primary concern among cancer survivors,^10^ remains a critical unmet need.^11^ While transient or low‐level FCR is normal and adaptive, persistent and excessive FCR can have debilitating effects, including heightened vigilance, chronic worry, and persistent body symptom awareness,^12^ leading to decreased treatment adherence,^13^ increased healthcare costs,^14^ and significantly reduced quality of life. Furthermore, the severity of FCR does not necessarily decrease over time, especially without appropriate intervention.^15^, ^16^

Hope theory posits that hope, a central concept in positive psychology, enables individuals to cope more effectively with adverse stimuli and maintain positive future expectations.^17^, ^18^ The level of hope plays a crucial role in patients' understanding of their disease and their response to it. Research indicates a correlation between hope levels and FCR in NSCLC patients,^19^ suggesting that higher hope levels can mitigate negative emotions like anxiety and pain, thus reducing FCR.

Social support, defined as the network of close relatives and friends available to assist patients during illness, provides crucial emotional and financial support.^20^ It encourages patients to accept their condition and pursue active treatment. Social support sources are diverse, encompassing family, colleagues, friends, and social groups. A supportive social environment can enhance patients' survival desire and psychological state,^21^ elevating their hope levels and promoting proactive measures to mitigate negative emotions, thereby effectively reducing social dysfunction occurrences.^22^

Restricted cubic splines (RCS) are an advanced smoothing technique in statistical modeling, particularly adept at handling data with significant variations in certain regions.^23^ This technique, crucial in biostatistics and epidemiology, models nonlinear relationships between exposure and risk. Its application extends to finance, engineering, and other data analysis domains.

In summary, despite significant improvements in the detection rate and surgical treatment outcomes of early‐stage lung cancer in recent years, patients with lung cancer report higher FCR scores compared to those with other tumors. For early‐stage NSCLC patients, even though the risk of postoperative recurrence and metastasis is relatively reduced, the level of FCR does not diminish. These patients commonly experience severe negative emotions, and both low levels of hope and serious social functional impairments increase the complexity of clinical care. Combining these factors can help improve the prognosis of early‐stage NSCLC patients. Traditional studies have mostly presented the correlation between these three aspects and modeled them through linear regression, representing the combined results of data and effects. This approach can somewhat reflect the factors influencing FCR, but since the dose–response relationship often exhibits nonlinearity, accurately quantifying this nonlinear relationship is crucial for effectively changing the FCR status of patients. Therefore, this study adopts a RCS model to explore the relationship between FCR, levels of hope, and social support, and conducts subgroup analyses. This provides a reference for preventing FCR in early‐stage NSCLC patients and for determining the intensity of interventions through nonlinear dose–response relationships.

## 2 SUBJECTS AND METHODS

### 2.1 Study subjects

Using a convenience sampling method, we selected 677 patients who underwent surgical treatment for early‐stage NSCLC at the Cancer Hospital of the Chinese Academy of Medical Sciences between January 2022 and August 2023. Inclusion criteria were: (1) aged ≥18 years; (2) intraoperative frozen pathology confirming NSCLC; (3) pathological staging of TNM IA‐IIB; (4) informed consent and willingness to participate in the survey. Exclusion criteria included: (1) presence of other severe physical or psychological diseases; (2) cognitive impairment; (3) language communication or literacy difficulties.

The study was independently approved by the Ethics committee of Cancer Hospital Chinese Academy of Medical Sciences (Approval No. 15‐032/959).

### 2.2 Research methods

#### 2.2.1 Survey tools

##### General information questionnaire

Designed by the researchers, it includes general demographic and clinical information of the patients, such as age, gender, number of episodes, duration of disease, number of lesions, education level, marital status, family monthly income per capita, method of medical expense payment, pathological staging, smoking history, type of surgery, and whether they have children.

##### Fear of Progression Questionnaire‐Short Form (FoP‐Q‐SF)

Originally developed by Mehnert et al.^24^ and translated into Chinese by Wu et al.,^25^ this unidimensional scale consists of 12 items, with a score range of 12–60. A score >34 indicates psychological dysfunction, with at least 50% of items scored 4 or higher indicating moderate fear of recurrence (FCR), and at least 75% scoring 4 or higher indicating severe FCR. It uses a Likert 5‐point scale from “never” to “always,” with higher scores indicating stronger fear. The Chinese version's item correlation coefficients range from 0.578 to 0.712, and its Cronbach's alpha is 0.883, indicating good reliability and validity.

##### Herth Hope Index (HHI)

Developed by Herth^26^ in the United States and translated and revised by Chinese scholar Zhao Haiping,^27^ it is commonly used to measure the level of hope in cancer patients. The scale includes three subscales: positive attitude towards reality and the future (4 items), active engagement (4 items), and maintaining close relationships with others (4 items), totaling 12 items. It uses a Likert 4‐point scale, and the total score ranges from 12 to 48, with higher scores indicating higher levels of hope. Hope levels are categorized as low (12–23), medium (24–35), and high (36–48). The Herth Index has shown good reliability, validity, and feasibility, with a test–retest reliability of 0.92, internal consistency reliability alpha of 0.87, and structural validity of 0.85.

##### Social Support Rating Scale (SSRS)

Developed by Xiao et al.,^28^ this scale has 10 items divided into three dimensions: objective support (3 items), subjective support (4 items), and social support utilization (3 items). Except for items 6 and 7, the rest use a 4‐point scoring method, with higher scores indicating better social support. The scale has shown good reliability and validity, with a test–retest reliability of 0.92 and item consistency between 0.89 and 0.91.

#### 2.2.2 Survey method

The survey was conducted using electronic questionnaires, distributed to postoperative early‐stage NSCLC patients who met the inclusion criteria. Data collectors received online training before the survey, including the purpose and significance of the study, questionnaire and scale content, data collection methods, and precautions. Before the survey, each data collector explained the purpose and significance of the study to the respondents using a consistent teaching language, obtained informed consent, and then distributed the questionnaire. To avoid interference between patients in the same ward, patients completed the questionnaire in a separate quiet room, with guidance on how to fill it out. Patients with poor vision had their questionnaires completed by their caregivers. A total of 715 questionnaires were distributed, of which 18 were incompletely filled, 12 had incomplete medical record numbers, and 8 had discrepancies between the medical record number and the patient's name. Ultimately, 677 valid questionnaires were collected, yielding an effective recovery rate of 94.7%. After retrieving the questionnaires, two people cross‐checked and supplemented the clinical data in the hospital's HIS information system to ensure accuracy.

#### 2.2.3 Statistical methods

The study used R4.1.2 for statistical analysis. Continuous variables were described using mean ± standard deviation, and comparisons between groups were made using *t*‐tests or ANOVA. The FCR was dichotomized into compensated and decompensated states for analysis. Predictors of binary variables were analyzed using logistic regression, with variables showing statistical significance in univariate logistic regression further included in multivariate logistic regression to identify independent risk factors. RCS regression was used to analyze the compensation of FCR, with four nodes based on previous literature, and the relative risk OR visualized. An OR >1 indicates a risk factor, OR <1 indicates a protective factor, and a 95% confidence interval crossing OR = 1 indicates no statistical significance.

## 3 RESULTS

### 3.1 Demographic characteristics

As shown in Table 1, this study included 677 postoperative early‐stage lung cancer (TNM IA‐IIB) patients. Among them, 256 were male (38%) with a FCR index of 28.36 ± 9.51, and 421 were female (62%) with a FCR index of 30.73 ± 8.74. Female patients exhibited significantly higher FCR than male patients (*p* = 0.001). Patients aged 18–35 years (8%) had a FCR index of 32.46 ± 8.113; those aged 36–60 years (60%) had an index of 30.97 ± 8.994; and those older than 60 years (31%) had an index of 26.92 ± 8.901, indicating that younger patients showed significantly higher FCR than older patients. Patients with a monthly income of less than 3000 yuan (17%) had a FCR index of 31.53 ± 10.07; those earning 3000–5000 yuan (33%) had an index of 30.84 ± 9.132; and those earning 5001–10,000 yuan (30%) had an index of 28.45 ± 8.689, showing that patients with lower income had significantly higher FCR than those with higher income.

**TABLE 1:** Baseline characteristics and variance analysis (*N* = 677).

Variables	Number	Percentage	Mean	SD	*t*/*F*	*p*
Sex						
Male	256	38%	28.36	9.505	10.944	0.001
Female	421	62%	30.73	8.743		
Age						
18–35	57	8%	32.46	8.113	17.143	0
36–60	409	60%	30.97	8.994		
>60	211	31%	26.92	8.901		
Number of episodes						
Initial onset	651	96%	29.92	9.035	1.662	0.198
Recurrence	26	4%	27.58	10.659		
Course classification						
0–12 months	505	75%	29.66	8.959	0.79	0.454
13–36 months	120	18%	29.9	9.788		
>36 months	52	8%	31.33	8.92		
Number of lesions						
Single	358	53%	29.18	9.378	3.941	0.048
Multiple	319	47%	30.57	8.745		
Surgical modality						
Thoracotomy	9	1%	29.33	10.665	0.027	0.868
Thoracoscopy	668	99%	29.84	9.091		
Smoking						
Yes	110	16%	28.43	10.781	3.141	0.077
No	567	84%	30.11	8.727		
Education level						
Junior high school or below	150	22%	28.48	10.74	2.431	0.089
High school graduate	141	21%	29.72	9.252		
College graduate or above	386	57%	30.4	8.292		
Ethnicity						
Han nationality	628	93%	29.71	9.036	1.541	0.215
Ethnic minorities	49	7%	31.39	9.916		
Religion						
Yes	33	5%	31.39	11.9	1.02	0.313
No	644	95%	29.75	8.943		
Place of residence						
City	491	73%	29.19	8.721	4.953	0.007
Villages and towns	146	22%	31.86	9.195		
Rural	40	6%	30.35	12.095		
Occupation						
Worker	57	8%	29.88	10.172	1.71	0.116
Technical personnel	77	11%	30.95	8.18		
Farmer	50	7%	29.96	12.108		
Businessmen or self‐employed workers	41	6%	30.66	8.912		
Administrative staff	126	19%	31.45	8.129		
Health care workers	34	5%	29.97	8.48		
Unemployed or retired	292	43%	28.68	8.952		
Monthly income						
<3000	114	17%	31.53	10.07	4.452	0.004
3000–5000	226	33%	30.84	9.132		
5001–10,000	200	30%	28.45	8.689		
>10,000	137	20%	28.78	8.446		
Medical payment method						
Own expense	31	5%	30.55	8.644	0.633	0.532
Public expense	54	8%	28.57	8.058		
Medical insurance	592	87%	29.91	9.221		
Type of pathology						
Squamous cell carcinoma	34	5%	29.21	10.327	0.184	0.832
Adenocarcinoma	641	95%	29.88	9.048		
Large cell carcinoma	2	0%	27	9.899		
T stage						
0	98	14%	30.57	7.93	0.969	0.407
1	506	75%	29.86	9.275		
2	66	10%	29.03	9.293		
3	7	1%	25.29	10.339		
N stage						
0	667	99%	29.88	9.119	0.983	0.322
1	10	1%	27	8.014		
Preoperative tumor markers						
Normal or unchecked	520	77%	30.16	8.933	2.892	0.089
Abnormal	157	23%	28.75	9.601		
Family history						
Yes	178	26%	30.23	9.505	0.459	0.498
No	499	74%	29.69	8.964		
Marital status						
Unmarried	28	4%	32.71	9.633	1.494	0.225
Married	618	91%	29.73	9.071		
Divorced	31	5%	29.32	9.167		
Whether you have children						
Yes	635	94%	29.64	9.099	4.582	0.033
No	42	6%	32.74	8.793		
Whether the child is an adult						
The children are all adults	488	72%	28.94	9.279	5.872	0.001
Children are partially adults	30	4%	31.6	8.52		
All underage	117	17%	32.08	7.988		
Childless	42	6%	32.74	8.793		
Living alone						
Yes	176	26%	29.95	9.338	0.038	0.845
No	501	74%	29.79	9.031		
Combined with the underlying disease						
Yes	232	34%	29.06	8.901	2.548	0.111
No	445	66%	30.24	9.193		
Chemotherapy						
Yes	34	5%	32.68	9.464	3.504	0.062
No	643	95%	29.68	9.068		
Radiotherapy						
Yes	16	2%	34.44	9.811	4.211	0.041
No	661	98%	29.72	9.066		
Herth Hope Index						
≤35	88	13%	35.02	11.03	34.489	0
>35	589	87%	29.06	8.525		
Social Support Rating Scale						
≤40	114	17%	33.62	10.221	24.577	0
>40	563	84%	29.07	8.672		

### 3.2 Univariate and multivariate logistic regression

As indicated in Table 2, variables showing statistical significance in variance analysis were included in the univariate regression analysis. It was found that being female and having underage children were significant risk factors for FCR. Being over 60 years of age, having a monthly income over 5000 yuan, and having high levels of hope and social support were significant protective factors. The multivariate logistic regression included variables that were statistically significant in the univariate logistic regression, identifying age (OR = 0.392, 95% CI 0.205–0.750), monthly income (OR = 0.016, 95% CI 0.315–0.886), hope level (OR = 0.305, 95% CI 0.187–0.496), and social support (OR = 0.584, 95% CI 0.375–0.908) as independent factors influencing FCR.

**TABLE 2:** Logistic regression.

	Univariate logistic regression				Multivariate logistic regression			
Variables	*p* value	OR	95%LCI	95%UCI	*p* value	OR	95%LCI	95%UCI
Sex								
Male								
Female	0.017	1.504	1.074	2.107	0.084	1.368	0.959	1.951
Age	0				0			
18–35								
36–60	0.803	0.931	0.529	1.638	0.772	0.917	0.509	1.652
<60	0.007	0.424	0.228	0.788	0.005	0.392	0.205	0.75
Number of lesions								
A single								
Multiple	0.085	1.324	0.962	1.822				
Place of residence	0.169							
City								
Villages and towns	0.093	1.388	0.946	2.036				
Rural	0.275	1.445	0.746	2.798				
Monthly income	0.022				0.018			
<3000								
3000–5000	0.571	0.876	0.553	1.387	0.839	0.951	0.585	1.547
5001–10,000	0.01	0.527	0.324	0.857	0.016	0.528	0.315	0.886
>10,000	0.063	0.609	0.361	1.026	0.082	0.612	0.352	1.064
Whether you have children								
Yes								
No	0.532	1.228	0.645	2.339				
Whether the child is an adult	0.059							
The children are all adults								
Children are partially adults	0.497	1.305	0.606	2.809				
All underage	0.008	1.741	1.152	2.631				
Childless	0.326	1.387	0.723	2.661				
Radiotherapy								
Yes								
No	0.17	0.499	0.185	1.347				
Herth Hope Index								
≤35								
>35	0	0.295	0.186	0.467	0	0.305	0.187	0.496
Social Support Rating Scale								
≤40								
>40	0.001	0.497	0.33	0.748	0.017	0.584	0.375	0.908

### 3.3 RCS analysis of the hope level and social support

**FIGURE 1:**
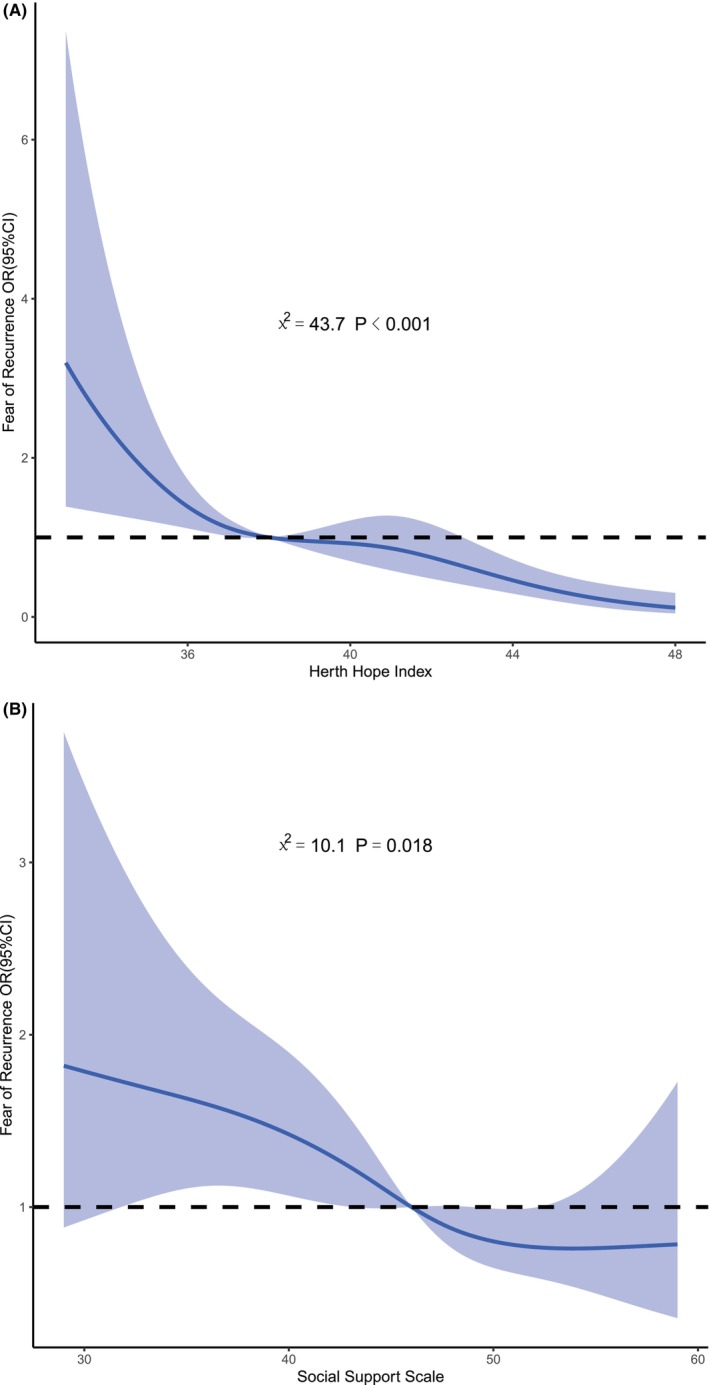
(A) Restricted cubic spline of fear of recurrence by the hope level. (B) Restricted cubic spline of fear of recurrence by social support.

After adjusting for age, gender, and income level, the RCS analysis method was used to analyze the nonlinear effects of various factors on FCR, determining if there were threshold or saturation effects. Figure 1 showed a significant nonlinear dose–response relationship between FCR and hope level (nonlinear *χ* ^2^ = 43.7, *p* < 0.001). With increasing hope levels, the risk of decompensated FCR gradually decreased and turned from a risk factor to a protective factor at a hope index of 38, with no significant saturation effect observed. Figure 1B demonstrated a significant nonlinear dose–response relationship between FCR and social support (nonlinear *χ* ^2^ = 10.1, *p* = 0.018). As social support increased, the risk of decompensated FCR gradually decreased and turned from a risk factor to a protective factor at around a social support score of 45. Saturation was reached at about 50, meaning further increases in social support did not significantly improve the risk of FCR.

### 3.4 RCS analysis in subgroups

**FIGURE 2:**
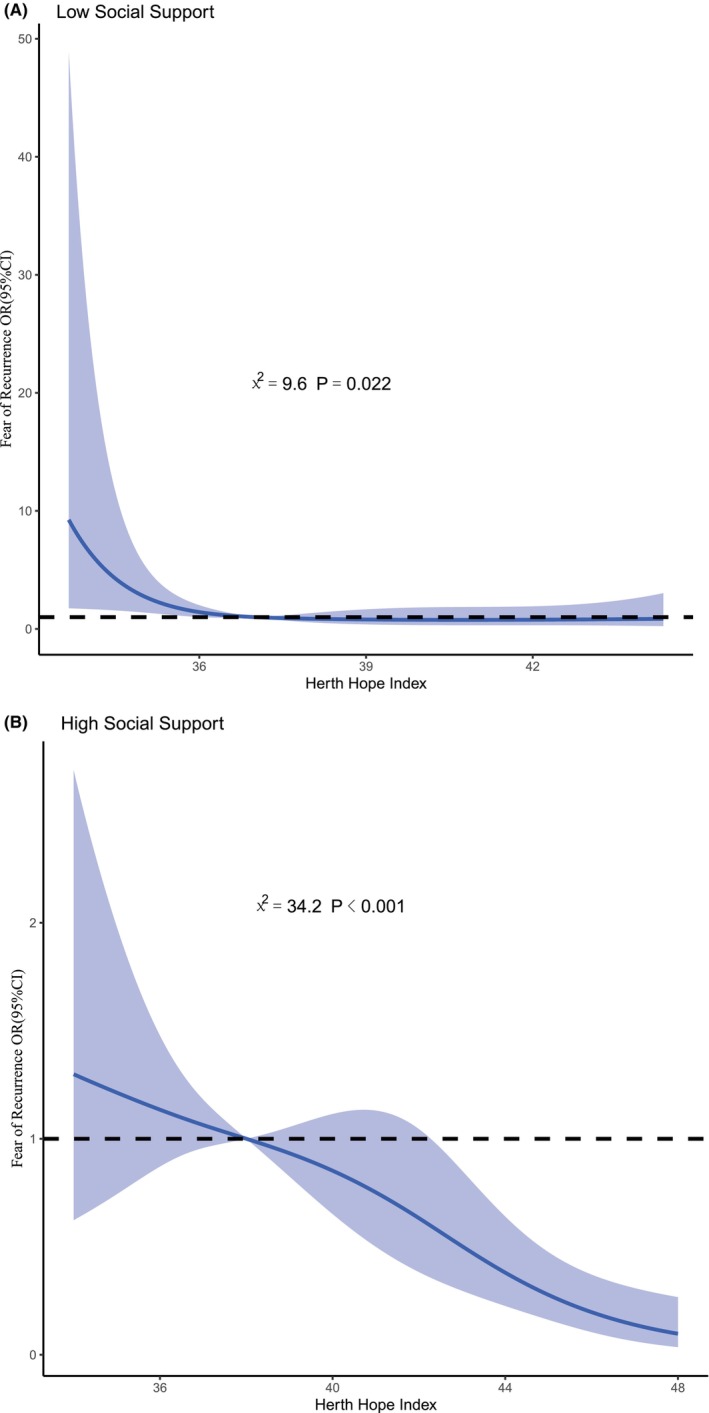
(A) Restricted cubic spline of fear of recurrence by the hope level in the low social support group. (B) Restricted cubic spline of fear of recurrence by the hope level in the high social support group.

Social support was divided into the low (≤40) and high (>40) groups for subgroup RCS analysis. Figure 2A in the low social support group showed a significant nonlinear dose–response relationship between FCR and hope level (nonlinear *χ* ^2^ = 9.6, *p* = 0.022). As the hope level increased, the risk of FCR gradually decreased, but reached saturation at about 36, and further increases in the hope level did not reduce the risk of decompensated FCR. Figure 2B in the high social support group showed a significant nonlinear dose–response relationship between FCR and hope level (nonlinear *χ* ^2^ = 34.2, *p* < 0.001). As the hope level increased, the risk of decompensated FCR gradually decreased, turning from a risk factor to a protective factor at a hope level of 38, with no apparent saturation effect.

## 4 DISCUSSION

### 4.1 Interaction between fear of recurrence and hope level

Our findings highlight a pivotal nonlinear dose–response relationship between FCR and hope levels. We observed a notable decrease in the risk of decompensated FCR with increasing hope levels. This aligns with existing literature,^29^ suggesting that elevated hope positively impacts patient outlook and treatment engagement. Interestingly, a hope index of 38 marks a threshold where hope transitions from a risk to a protective factor, effectively diminishing FCR without notable saturation. These insights underscore the importance of fostering hope in patients, potentially through patient education on disease prognosis, treatment advances, and encouraging peer interactions.

### 4.2 Fear of recurrence and social support

The study underscores a significant nonlinear relationship between FCR and social support. Enhanced social support appears to mitigate adverse emotional responses and FCR, as supported by Qian et al.^30^ We found that high social support (scores around 45) alters FCR dynamics, transforming it from a risk to a protective factor. However, there seems to be a saturation point around a score of 50, beyond which additional social support does not yield further benefits in reducing FCR. This finding suggests a tailored approach where medical professionals should intensify social support for patients with low levels, while for those with high support, the focus should shift to addressing other unmet needs to mitigate FCR.

### 4.3 Low social support group

In patients with lower social support, the study revealed a significant nonlinear relationship between hope levels and FCR. Increasing hope levels can satisfy and empower patients, enhancing their resilience against the disease.^31^ However, a saturation point at a hope level of approximately 36 was observed, beyond which no additional reduction in FCR risk occurs. This indicates that in the context of low social support, merely increasing hope is insufficient. Medical professionals should prioritize enhancing the social support network alongside hope elevation efforts.

### 4.4 High social support group

Among patients with higher levels of social support, increasing hope levels further reduces the risk of FCR. Notably, a hope level of 38 or higher creates a synergistic effect, continuously lowering FCR without apparent saturation. This suggests that in patients with substantial social support, efforts to elevate hope above this threshold could be particularly effective in minimizing FCR, facilitating a smoother transition back to normalcy.

### 4.5 Clinical implications

FCR is a widespread concern impacting a majority of cancer survivors and necessitates attention from healthcare providers and policymakers. This research leverages a RCS model to discern the nonlinear dynamics between hope, social support, and FCR. It underscores the need for medical staff and family members to prioritize increasing social support as a foundational step towards boosting hope levels and reducing FCR. The study provides valuable metrics for hope and social support that are instrumental in reducing FCR, guiding nursing managers in formulating targeted intervention strategies.

### 4.6 Strengths

(1) The focus on early‐stage NSCLC patients, who typically experience heightened FCR; (2) application of the RCS model delineates clear numerical benchmarks for the nonlinear interactions between hope levels, social support, and FCR reduction, aiding in the development of effective interventions; (3) the study enhances awareness among medical professionals and families that augmenting social support is essential for optimally reducing FCR through increased hope levels.

### 4.7 Limitations

The limitations of our study are: (1) As a single‐center cross‐sectional study, ' broader, multicenter research is warranted; (2) while several relevant medical and demographic factors were identified, other pertinent factors may remain unaccounted for; and (3) the potential for residual confounding persists despite adjustments for known confounders.

## 5 CONCLUSION

Our findings reveal that: (1) with the hope index exceeding 38, FCR diminishes as hope increases, establishing itself as a protective factor without saturation; (2) social support around 45 points leads to a reduction in FCR, reaching saturation at about 50 points; and (3) among those with low social support, FCR plateaus when hope levels approximate 36. These observations suggest that social support functions as a catalyst in modulating the impact of hope on FCR. Enhancing social support is a critical first step before elevating hope levels to effectively reduce FCR.

## AUTHOR CONTRIBUTIONS

**Man Liu:** Data curation (equal); investigation (equal); writing – original draft (equal). **Lu Liu:** Data curation (equal); funding acquisition (equal); investigation (equal). **Zhuoheng Lv:** Data curation (equal); methodology (equal); validation (equal). **Yousheng Mao:** Resources (equal); supervision (equal). **Yan Liu:** Resources (equal); writing – review and editing (equal).

## FUNDING INFORMATION

Supported by Beijing Hope Run Special Fund of Cancer Foundation of China (LC2022C05).

## CONFLICT OF INTEREST STATEMENT

The authors declare that they have no conflicts of interests.

## ETHICS STATEMENT

All procedures performed in this study were conducted following the Declaration of Helsinki (as revised in 2013). All patients provided written informed consent before enrollment.

## CONSENT

Informed consent was obtained from all individual participants included in the study.

## Data Availability

Data sharing is not applicable to this article as no new data were created or analyzed in this study.
